# The artificial intelligence and machine learning in lung cancer immunotherapy

**DOI:** 10.1186/s13045-023-01456-y

**Published:** 2023-05-24

**Authors:** Qing Gao, Luyu Yang, Mingjun Lu, Renjing Jin, Huan Ye, Teng Ma

**Affiliations:** 1 Cancer Research Center, Beijing Chest Hospital, Capital Medical University, Beijing Tuberculosis and Thoracic Tumor Research Institute, Beijing, 101149 China; 2 Department of Respiratory and Critical Care Medicine, Beijing Chest Hospital, Capital Medical University, Beijing Tuberculosis and Thoracic Tumor Institute, Beijing, 101149 China

## Abstract

Since the past decades, more lung cancer patients have been experiencing lasting benefits from immunotherapy. It is imperative to accurately and intelligently select appropriate patients for immunotherapy or predict the immunotherapy efficacy. In recent years, machine learning (ML)-based artificial intelligence (AI) was developed in the area of medical-industrial convergence. AI can help model and predict medical information. A growing number of studies have combined radiology, pathology, genomics, proteomics data in order to predict the expression levels of programmed death-ligand 1 (PD-L1), tumor mutation burden (TMB) and tumor microenvironment (TME) in cancer patients or predict the likelihood of immunotherapy benefits and side effects. Finally, with the advancement of AI and ML, it is believed that "digital biopsy" can replace the traditional single assessment method to benefit more cancer patients and help clinical decision-making in the future. In this review, the applications of AI in PD-L1/TMB prediction, TME prediction and lung cancer immunotherapy are discussed.

## Introduction

Lung cancer is the deadliest cancer type in China and one of the deadliest cancers in the world [1]. Currently, immunotherapy has shown promising results in lung cancer patients. However, the objective response rates vary considerably among patients. Therefore, it is important to accurately identify lung cancer patients sensitive to immunotherapy.

AI has become increasingly relevant to all aspects of human life due to the development of statistical methodology and big data science. AI focuses on simulating human intelligence, thinking, and reasoning models to solve problems, provide decisions and automate labor. As a subset of AI, ML is defined as a method of analyzing a large amount of sample data with a target task and then, parsing that data into predictive models and clustering by itself, which is then analyzed by the computer [2]. AI specifically refers to the concept of a "thinking machine," emphasizing the computer's ability to make independent decisions, while ML refers to a "learning machine," which can complete tasks without explicit programming instructions by inputting data and implementing algorithms to create a computing framework [3]. Deep learning (DL) algorithms, a subset of ML, are AI-driven algorithms that can profoundly impact biomedical research, personalized medicine, and precision medicine [4]. By analyzing genomics, pathomics, imaging, and other biological data with computers, mathematical modeling, and applying it to clinical and scientific research, ML is a method for discovering new things about patients. It has become a hot topic of development these days to cross-fertilize medicine and artificial intelligence [5].

PD-L1 and programmed cell death protein 1 (PD-1)-based lung cancer immunotherapy is the most successful immune checkpoint blockade (ICB) therapy. The tumor intrinsic characteristics such as TMB and TME also affect the immunotherapy efficacy. In this review, we comprehensively examine the advances in AI and ML-driven applications in lung cancer immunotherapy.

## AI and ML

### The conceptions and applications of AI and ML

The training process in ML can be categorized into three main types: supervised learning, unsupervised learning (UL), and semi-supervised learning (SSL). Supervised learning involves using labeled data to train the model. Some typical classification and regression models for supervised learning include k-nearest neighbor (KNN), linear regression (LR), support vector machine (SVM), decision trees (DT), and random forests (RF). In contrast, UL does not involve labeling the data, and SSL combines labeled and unlabeled data. The labeling process can be time-consuming and labor-intensive, but it can result in better performance of the models since they have been externally validated [6]. Clustering models are common UL algorithms, including k-means clustering, hierarchical clustering, and principal component analysis (PCA).

Overall, the choice of learning type depends on the data and the task at hand. Supervised learning is suitable for tasks that require predicting a specific output from input data with known labels, while UL is used to discover hidden patterns and structures in data. SSL is used when there is a limited amount of labeled data available.

DL is a subset of ML that utilizes neural networks as its fundamental algorithm. Unlike traditional ML, DL does not rely on domain experts to manually engineer features. Instead, it mimics the iterative transmission of information in the human brain by using neural networks to automatically learn representations of data. During training, the algorithm adjusts its parameters to optimize the model and produce the best output. DL has found applications in various fields, including computer vision (CV), natural language processing (NLP), and speech recognition.

In the medical field, NLP has been applied to various scenarios, including simple internet-based AI consultations, information extraction from electronic medical records, and automatic case writing [7]. Additionally, CV has been extensively employed for medical image recognition in areas such as computer tomography (CT), X-ray, positron emission tomography/computer tomography (PET/CT), and immunohistochemistry (IHC) [8]. Many reported models for predicting lung cancer risk utilize supervised ML, such as artificial neural network (ANN), DT, RF, SVM and Bayesian classification.

### The history of AI and ML

**Fig. 1:**
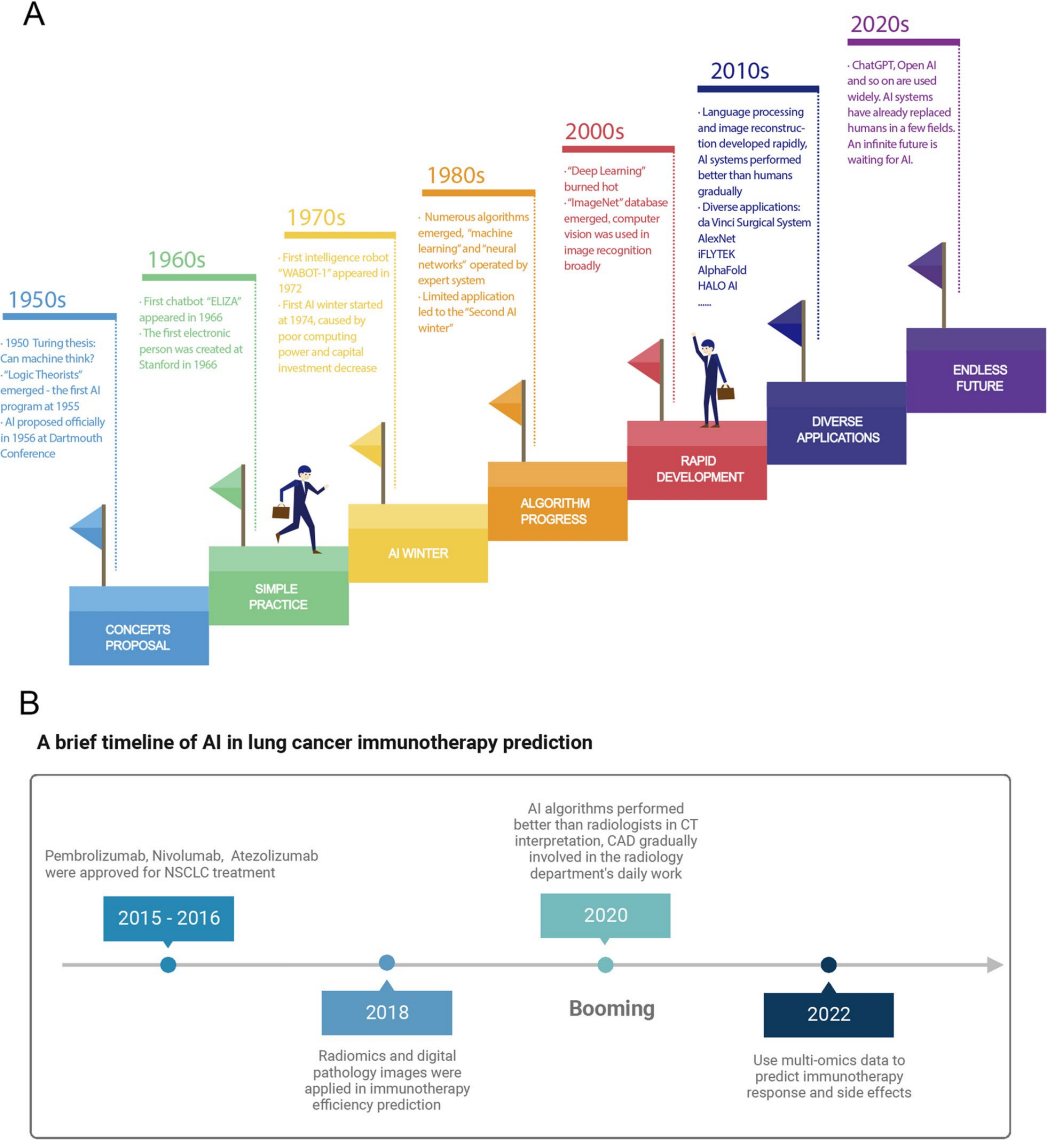
**A** The Development History of Artificial Intelligence and Machine Learning. Timeline of the development history of artificial intelligence and it’s milestone events of applications in medical care. **B** A brief timeline of Al in lung cancer immunotherapy prediction. Abbreviation: NSCLC: Non-small cell lung cancer; CAD: Computer-aided diagnosis. Figure 1B was created with BioRender.com

The concept of AI was officially proposed at the Dartmouth Conference in 1956 (Fig. 1). Scientists want to create machines that can mimic human intelligence [3].

During the early days of the 1960s, computers’ operation relied on the “expert system”, which refers to a large number of manual interpretation rules input by experts, forming a knowledge database [3].

In the 1970s, the limitation of the development of hardware equipment led to insufficient computing power, making it difficult to calculate large-scale data and complex missions. As a result, capital investment gradually decreased, and the evolvement of AI reached a stalemate, entering the “AI winter” period in history [3].

Until the 1980s, the concepts of ML and neural networks emerged. Canadian scholar Geoffrey Hinton improved the traditional perceptual network structure, coupled with the invention of back propagation and the extensive application of statistical principles, AI gained the ability to solve practical problems and gradually had commercial value [9]. Concurrently, AI has also developed in the fields of life sciences and medicine. Additionally, the development of the Internet promoted progress in NLP and data mining greatly.

In 2009, Li Feifei presented the ImageNet database for the first time as an academic poster at the Conference on Computer Vision and Pattern Recognition (CVPR), which expanded the types of samples that can be used for AI training, promoting the process of CV and image recognition greatly. With the advent of the Big Data era and the development of computer hardware, the concept of DL was proposed and emerged, which led to the development of convolutional neural networks (CNN) and deep neural networks (DNN). Since then, AI has entered a peak period of research and development, becoming well-known to the public [3].

Additionally, bioinformatics and semantic analysis technologies were also developed rapidly. In 2015, Canada’s DNA sequencing data enabled the identification of mutation sites and therapeutic targets, thus providing personalized treatment plans for patients. Furthermore, a speech recognition assistant developed by iFlytek and Tsinghua University was able to analyze patients’ conditions and provide auxiliary diagnoses [3].

Later on, with the development of big data, the evolution of ML algorithms, and the improvement of model prediction performance and generalization capabilities, AI is increasingly being applied in the field of biomedicine, including protein structure and function prediction, nucleotide sequencing analysis, drug characteristics, speech recognition and network consultation, auxiliary diagnosis mapping, risk prediction modeling, robot-assisted surgery and other fields [3] (Fig. 1).

However, individualized diagnoses and therapeutic strategies, such as early screening and diagnosis, functional visualization of key molecular events and targeted drugs, are still imperative for lung cancer treatment. New technologies such as tumor-assisted diagnosis combined with AI, analysis of molecular pathology information, prediction of tumor invasion and treatment resistance, and multi-omics fusion modeling to predict treatment outcomes and prognosis are providing new ideas and opportunities for clinicians.

## AI in lung cancer PD-L1 and TMB prediction

### The role of PD-L1 and TMB in lung cancer immunotherapy

Lung cancer is one of the most common cancers in the world with the highest mortality rate (1). Immune checkpoint inhibitors (ICIs) targeting PD-1, cytotoxic T-lymphocyte-associated protein 4 (CTLA-4) and PD-L1 have been widely developed and have shown good efficacy in treating non-small cell lung cancer (NSCLC). However, only about 30% of patients are eligible for treatment. Immune-related adverse events (irAEs) always occurred with patients, too. Traditional inspection methods are often limited in their ability to achieve expected benefits [10, 11].

PD-L1 expression and TMB were the first clinically assessed biomarkers. TMB is the total number of non-synonymous somatic mutations per megabase in the coding region of the tumor genome, with a wide range of mutations [12]. High TMB is positively associated with more tumor-associated neoantigens and improved immunotherapeutic efficacy [12]. Whole exome sequencing (WES) is the gold standard for assessing TMB. Studies have shown that NSCLC patients with above-median WES mutations have longer overall survival (OS) [13]. TMB is commonly used as a pan-cancer biomarker to identify patients who may benefit from PD-1 therapy, as it is a surrogate for tumor neoepitope burden. In addition, the expert consensus on immunotherapy for lung cancer recommends the use of mutational landscapes to assess the efficacy of PD-L1 immunotherapy in NSCLC [11].

### The application of AI and ML in lung cancer PD-L1 and TMB

According to the National Comprehensive Cancer Network (NCCN) guidelines, the expression status of PD-L1 protein levels determined by IHC via biopsy is the sole clinically approved biomarker for the evaluation of ICI therapy [14]. Higher levels of PD-L1 expression are typically associated with more favorable immunotherapeutic outcomes [15], but this relationship is not necessarily positive [16].

PD-L1 values obtained through routine pathology reports are lacking in a definitive gold standard and are instead crude, subjective, and semi-quantitative [17], resulting from gene mutations and sampling site differences. These factors result in significant inter-observer variability and approximately 30% inconsistency in judgments around the cut-off point [18, 19].

Moreover, due to the highly invasive nature of puncture biopsies or surgical specimens, which are often sampled during a single visit, the results are susceptible to static tumor characteristics and intratumoral heterogeneity making them less effective in predicting the benefit of immunotherapy [20, 21]. Thus, it is crucial to develop noninvasive and robust methods for interpreting PD-L1 expression that can be reviewed multiple times during follow-up or to identify alternative biomarkers [12].

**Fig. 2:**
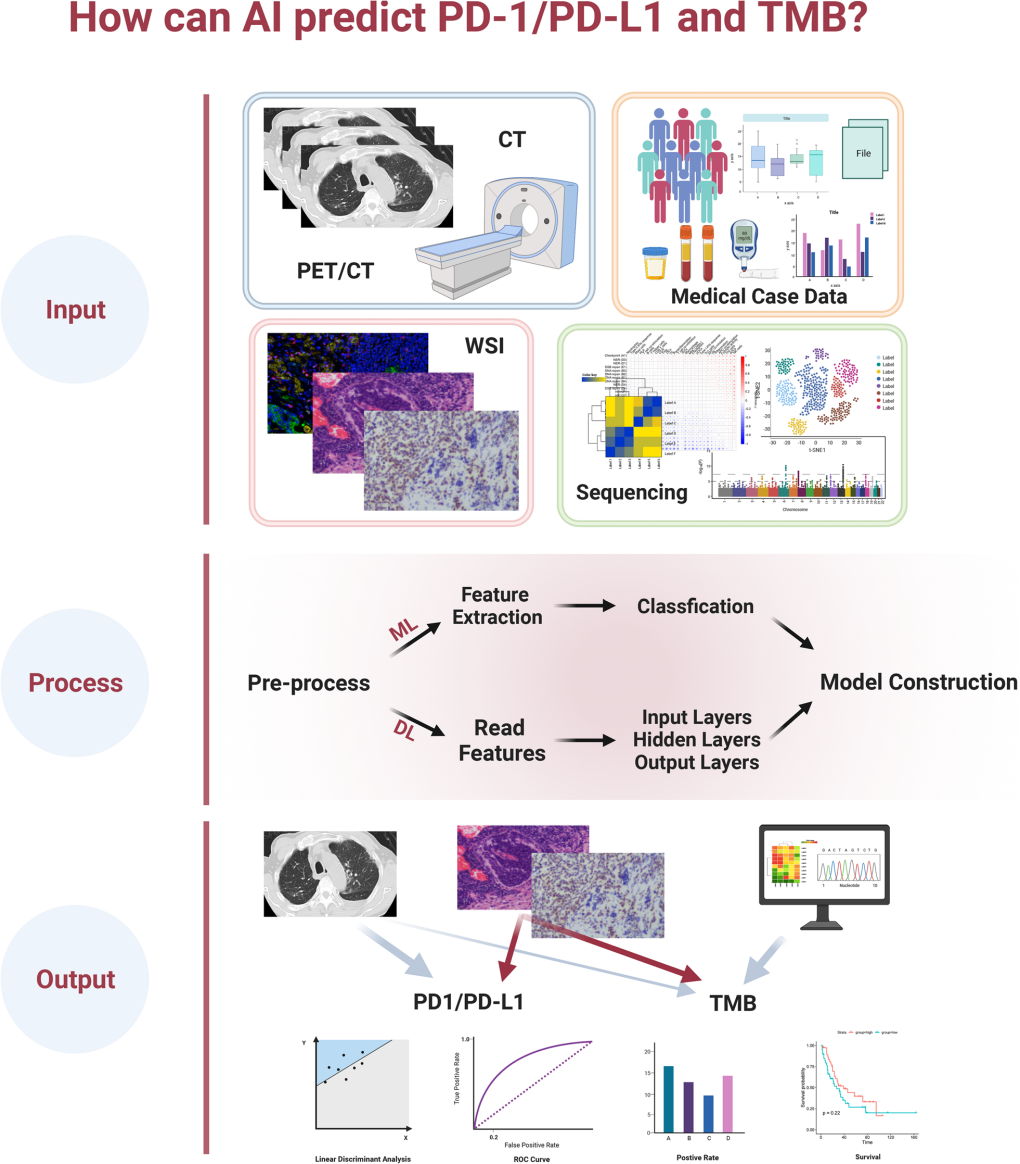
General process for AI-based PD-1/PD-L1 and TMB prediction. In the modeling process of machine learning, it generally goes through the process of feature collection, data preprocess, feature extraction, model establishment, performance evaluation, etc., and finally, obtains a prediction model. Abbreviation: WSI: Whole slide image. Created with BioRender.com. The heatmap was reprinted from *Mol Cancer*, Jin R, Liu B, Yu M, Song L, Gu M, Wang Z, et al. Profiling of DNA damage and repair pathways in small cell lung cancer reveals a suppressive role in the immune landscape. 2021;20(1):130, Copyright (2021) [80], licensed under CC BY 4.0 from Springer Nature

The accumulation of patient demographics, imaging, pathology images, laboratory data, medical history, sequencing data and other comprehensive information furnishes clinicians and statisticians with a substantial foundation of big data to analyze and identify the characteristics of people who benefit from ICIs. The utilization of ML to analyze multi-omics data for modeling and prediction efficiency and survival status has become the most promising development in precision medicine (Fig. 2) (Table 1).

**Table 1:** Machine learning algorithm predicts PD-L1, TMB, TME in lung cancer

Omics	Category	Task	Secondary task	Algorithm	Year	Description
Radiomics	PD-L1	Expression	Prognosis	RF	2020 [22]	Extracting image features from CT images to predict PD-L1 expression level and progression risk
Radiomics	PD-L1	Expression		SResCNN	2021 [14]	Using SResCNN to analyze PET/CT images and clinical data, using DLS score to predict PD-L1 expression
Radiomics	PD-L1	Expression		Logistic regression, RF	2020 [25]	Extracting features from CT, PET, and PET/CT images to model and predict the positive and high expression of PD-L1 simultaneously
Radiomics	PD-L1	Expression	Survive	DL	2020 [5]	Using deep learning to find CT image features to distinguish TMB expression and to predict survival in patients treated with ICIs
Pathomics	PD-L1/TMB	Expression	Treatment	ML	2023 [29]	Extraction of the tumor, mesenchymal, and TIL counts from HE-stained images for modeling and assessment of TMB and PD-L1 expression levels and efficacy prediction
Multi-omics	PD-L1	Treatment		ML	2022[30]	Combining sequencing data, IHC images, demographic data and laboratory data to predict the efficacy of immunotherapy
Multi-omics	PD-L1	Expression	Pneumonia	LCI-RPV	2023 [20]	The LCI-RPV model was developed to predict the ratio of PD-L1 expression to pneumonia by collecting CT images, CD274 counts and PD-L1 mRNA expression data
Multi-omics	TMB	Expression		ML	2022 [31]	Combining genomic and epigenetic data to predict TMB
Radiomics	TME	Prognosis	Treatment	ML	2020 [39]	Extracting PET/CT image features to ^+ ^distinguish groups who benefit from immunotherapy
Radiomics	TME	Expression	Treatment	ML	2022 [37]	Predicting TME by modeling PET/CT image features with CD8^+^T expression data to predict the immune status
Radiomics	TME	Expression	Prognosis	ML	2022 [38]	Extracting pGGO features from CT images combined with associated risk genes modelling to predict TME
Pathomics	TME/TIL	Expression	Prognosis	CNN	2018 [40]	Use CNN to analyze HE images in the database, model and predict TME and OS
Pathomics	TIL	Expression	Prognosis	CNN	2022 [41]	Development of I-score to predict clinical risk using CNN analysis of CD3^+^ T cell and CD8^+^T cell densities in WSI images
Pathomics	TME	Prognosis		CNN	2020 [42]	Improved boundary recognition for WSI images, extraction of spatial features modeling prognosis
Pathomics	TIL	Prognosis		Lunit SCOPE IO	2022 [43]	Segmentation and quantification of WSI images to build the model Lunit SCOPE IO analysis TIL
Multi-omics	TIL	Prognosis		Unsupervised clustering	2022 [44]	Extraction of TIME, patient survival data, SMG and CNV modeling to analyze TIL
Multi-omics	TME	Prognosis	Treatment	ML	2022 [45]	Screening gene combinations and modelling to predict OS and efficacy
Multi-omics	TME	Expression		K-means, SVM	2022 [46]	Screening, modeling, and predicting TIME of gene profiles using K-means and SVM

*PD-L1* Programmed Death Ligand 1, *TMB* Tumor Mutation Burden, *TME* Tumor Microenvironment, *CT* Computer Tomography, *RF* Random Forests, *SResCNN* Small Residual Product Network, *LightGBM* Light Gradient Boosting Machine, *DL* Deep Learning, *ML* Machine Learning, *ICI* Immune Checkpoint Inhibitor, *WSI* Whole Slide Image, *TIL* Tumor Infiltrating Lymphocyte, *CNN* Convolutional Neural Networks, *SVM* Support Vector Machine, *SMG* Significantly Mutated Gene, *CNV* Copy Number Variation, *TIME* Tumor Immune Microenvironment, *OS* Overall Survival, *pGGO* Pure Ground-Glass Opacity

#### Radiomics-based AI in PD-L1 and TMB prediction

Radiomics-based AI extracts subtle change features from noninvasive radiomic images, quantifying them based on the relationship between quantitative imaging and gene expression, and combining this with clinical data modeling to predict PD-L1 expression levels [22]. These AI systems can effectively avoid the invasive nature of biopsies and inter-tumor heterogeneity and provide unbiased and robust PD-L1 scores with greater clinical reference value [14].

Several ML algorithms based on PET/CT imaging have been utilized for feature extraction and modeling to forecast PD-L1 expression levels.  the In the three-variable linear discriminant model, metabolic parameter features from PET/CT images were extracted, achieving a sensitivity of 81% and a specificity of 82% in the test set [23]. Although these models are promising in predicting strong PD-L1 expression, distinguishing positive PD-L1 (PD-L1 > 1%) remains challenging. The small-residual-convolutional-network (SResCNN) was used to examine images and clinical data of PET/CT NSCLC patients, and a deep learning score (DLS) model was used to predict PD-L1 expression levels. This approach has shown significant improvements in predicting positive and negative patients with a receiver operating characteristic curve (ROC) = 0.82 and may serve as an alternative to IHC [14].

Although PET/CT can provide more image and parametric information, its high cost and technical requirements restrict its availability to many patients, making it difficult to gather PET/CT image data. But CT images are universal, easier to read and can provide comprehensive follow-up data. Therefore, there are also many studies that focus on predicting PD-L1 expression based on CT images.

Vaidya et al. [22] utilized the texture and quantitative vascular tortuosity (QVT) as the training features in CT images of NSCLC patients and then, used an RF classifier to forecast progression risk in patients receiving PD-1/PD-L1 therapy. In another study of 125 NSCLC patients before treatment, the logistic regression model is utilized to predict PD-L1 expression with the best performance, with an AUC of 0.85. During the training process, researchers used a ridge regression-based recursive feature elimination approach to select valuable features by manually determining the tumor area and radiomics features [24].

Jiang et al. used a cohort of 399 patients with stage I-IV NSCLC to perform tumor segmentation on PET/CT images and selected primary lesions. They correlated the expression status of PD-L1 with the features of CT, PET, and PET/CT images to build prediction models. The results showed that both CT and PET/CT obtained good performance, and the prediction model derived from CT performed the best, reaching the score of AUCs at 0.97 and 0.80 for PD-L1 > 1% and PD-L1 > 50% prediction [25]. These results indicated that radiomic-based approaches can predict PD-L1 expression accurately by combining features. And it deserves further exploration for guiding PD-L1 examination during clinical immunotherapy.

Furthermore, combinatorial multi-omics approaches were also used. Chen et al*. *[20] collected CT images, CD274 counts, and PD-L1 mRNA expression data from NSCLC patients, and they developed an LCI-RPV model to predict the ratio of PD-L1 expression to pneumonia with an AUC of 0.7.

A TMBRB model was generated to distinguish the efficacy of ICIs in NSCLC patients by assessing the expression level of TMB [5].

In conclusion, AI has demonstrated potential in predicting PD-L1 expression in lung cancer patients in radiomics image analysis and modeling (Fig. 2). Combining multi-omics data may be the promising direction for its further improvement and performance.

#### Pathology-based AI in PD-L1 and TMB prediction

In addition to imaging data, pathological slice images can also be used to establish predictive models for PD-L1 and TMB [26].

Whole slide image (WSI) technology uses digital pathological scanning systems to convert traditional pathological slices into high-resolution images. The fragmented images are stitched together into a complete image by computers, solving the problems related to preservation, loss and image fading [27]. WSI also can perform preprocessing such as homogenization on digital images. Furthermore, this digital method has the advantages of high efficiency and is not limited by sequencing. However, it is still difficult to achieve uniform batch variation due to time differences, reagent differences, and staining method differences [28].

Currently, the WSI-based AI classification tool "HALO AI" has been developed and is widely used in scientific research experiments related to tumor immunity. HALO AI has undergone supervised ML training of pathologists' marked features and can automatically classify tissues or cells on the entire pathological image or evaluate tumor areas, stromal areas, non-tumor non-stromal areas, etc. HALO AI can generate visual feature reports quickly and efficiently.

Besides HALO AI, Rakaee et al*.* developed an automated method based on ML to evaluate the expression of TMB and PD-L1 by counting the tumor, stroma, and tumor infiltrating lymphocyte (TIL) cells in hematoxylin and eosin (HE) stained images. The clinical outcomes of NSCLC patients were then linked to construct the model. The results showed that the combination of TILs/PD-L1 (AUC = 0.77) or TMB/PD-L1 (AUC = 0.65) had a better ability to predict the response to ICI treatment than using single PD-L1 prediction, and this approach may be used for accurate treatment [29].

To resist the visual deviation, Liesbeth M Hondelink's group developed a tumor proportion score (TPS) algorithm based on DL using PD-L1 to predict the efficacy of immunotherapy. They used WSI image data training of patients diagnosed with stage IV NSCLC, and the results showed more than 75% consistency with the reference score and the judgment of the pathologist. This algorithm can be used as a scoring assistant [19].

Due to the correlation between PD-L1 score and immune benefit, most models based on pathological slides can predict the efficacy of immunotherapy or the survival time of patients via predicting PD-L1 and TMB.

#### Multi-omics-based AI in PD-L1 and TMB prediction

Byeon et al*. *[30] presented a model to anticipate the efficacy of immunotherapy, for instance, PD-1/PD-L1, by integrating demographic data, laboratory test data, sequencing data, and IHC images.

To predict TMB, scholars segregated adenocarcinoma patients from The Cancer Genome Atlas (TCGA) based on their TMB levels and utilized the differential mRNAs, miRNAs and Methylated CpG sites as prognostic features. They established a TMB prediction model using ML methods, which yielded an AUC of 0.895 in the validation cohort. This model can be validated using quantitative real-time-polymerase chain reaction (qRT-PCR), thus replacing traditional WES and circumventing certain conventional limitations [31]. Meanwhile, the RF classifier was used to train the model, and the number of frameshift mutations and other features are obtained by the public anti-PD-1 dataset. The results demonstrate that the integrated feature model's prediction performance is superior to that of a single TMB [32]. Such studies have shown that mutations in oncogenes relative to TMB levels disproportionately modulate anti-PD-1 responses. Perhaps while optimizing the algorithm, integrating other biomarkers is also an effective way to improve the performance of the model.

TMB is also independent of TME which includes quantitative values of TPS, stromal CD8^+^ TIL’s density, and stromal Foxp3^+^ TIL density, while smoking, serum CEA (sCEA), etc., may act as independent predictors of TMB [33].

## The application of AI and ML in lung cancer TME prediction

TME comprises a complex interaction of tumor cells, immune cells, cancer-associated fibroblasts (CAFs), signaling molecules and extracellular matrix components. The immunosuppressive TME in lung cancer has been shown to promote tumorigenesis [34, 35].

**Fig. 3:**
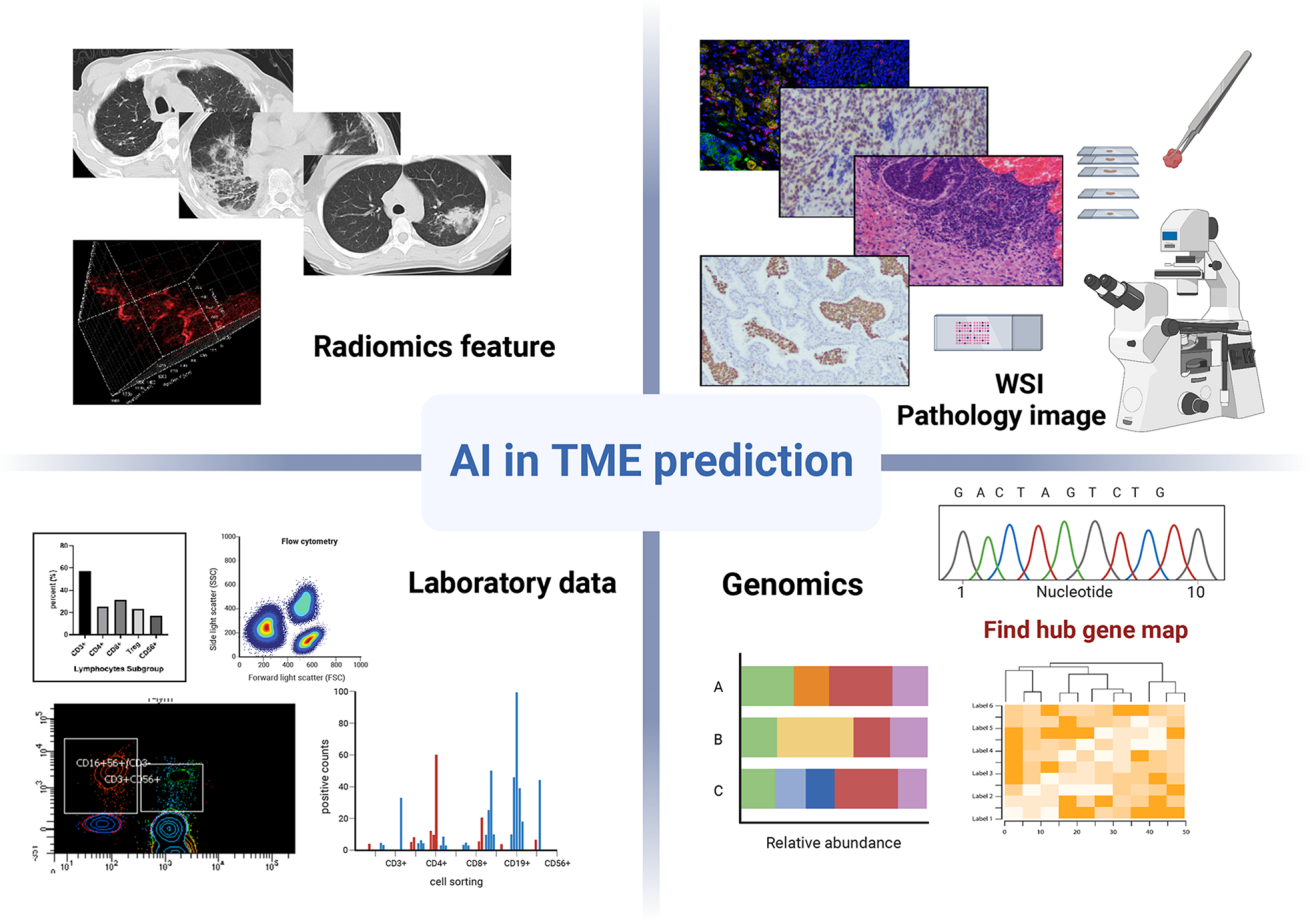
AI-based lung cancer TME prediction. Abbreviation: WSI: Whole slide image. Created with BioRender.com

WSI can facilitate TME evaluation. Most models can analyze morphological features of cells and structures in tissues by the processes such as image segmentation, feature extraction and scoring. The higher percentage of TIL in TME is a favorable prognostic factor for patients’ outcomes, whereas features such as angiogenesis are adverse prognostic factors [17]. Moreover, there is compelling evidence to suggest that the spatial distribution of lymphocytes in TME (central versus infiltrating margins) is a highly predictive factor of cancer prognosis [36]. Therefore, leveraging AI to score TME and TILs through image analysis and modeling has significant research value and broad application prospects in predicting the efficacy of tumor immunotherapy (Fig. 3) (Table 2).

**Table 2:** Summary of machine learning methods in lung cancer immunotherapy prediction

Material	Task	Secondary task	Algorithm
CT, PET/CT	Prognosis	Efficacy of immunotherapy	DT, BT, RF, SVM, GLM, ANN, CNN
Genomics	Treatment response	Survive	RF, MLP, unsupervised clustering
Proteomics	Survive		Iterative unsupervised machine learning
Microbiology	Survive	Treatment response	RF, MLP
Blood	Survive	Efficacy of immunotherapy	RF, MLP, SVM, elastic network, partial least squares discriminant analysis, Gaussian process classifier
Blood	irAE		ANN
Database	irAE		XGBoosted

*CT* Computer Tomography, *PET/CT* Positron emission tomography/Computer Tomography, *DT* Decision Trees, *BT* Boosting Tree, *RF* Random Forests, *SVM* Support Vector Machine, *GLM* Generalized Linear Model, *ANN* Artificial Neural Network, *CNN* Convolutional Neural Network, *MLP* Multilayer Perceptron, *XGBoosted* eXtreme Gradient Boosting

### Radiomics-based AI in TME prediction

In prior investigations about NSCLC, radiomics images have been implemented to anticipate alterations in tumor-infiltrating CD8^+^ T cell levels, with the aim of distinguishing patients who would benefit from PD-L1 therapy. Such endeavors have revealed the potential of radiomics in predicting TME [31].

Understanding the individual differences in TME can aid in the screening of populations who may respond to immunotherapy. Researchers have collected baseline PET/CT radiomics data and CD8 expression data from tumor specimens of 221 NSCLC patients. They employed ML models to predict the TME phenotype, thus ascertaining the immune status of NSCLC. This constitutes one of the several attempts to achieve noninvasive TME prediction through imaging-clinical joint models [37].

Presently, the utilization of CT scans for the early detection of lung cancer in high-risk groups is being widely advocated. Ground-glass opacity (GGO) has been identified as an imaging characteristic of early lung cancer. Although the corresponding pathological features do not meet surgical criteria, the diagnostic potential of GGO for early-stage lung cancer cannot be disregarded. Consequently, a team of researchers has constructed a 15-gene risk signature related to pure ground glass opacity (pGGO) through transcriptome analysis. They have utilized this signature to predict the prognosis of early-stage lung adenocarcinoma (LUAD) and to investigate the immune microenvironment of GGO. The predictive ability of this signature for patients with early-stage adenocarcinoma has been verified in TCGA and Gene Expression Omnibus (GEO) datasets [38].

Many studies of similar nature are currently emerging, and the algorithms and performance of the models are being continuously improved [39]. These studies substantiate the promising future of radiomics in TME prediction.

### Pathology-based AI in TME prediction

Researchers use both authentic cases and HE stained images in databases to train models. Previous research endeavors incorporated HE images of thirteen cancer types from TCGA in order to map TILs using CNN. Researchers try to elucidate the local spatial structure in TME and its association with OS [40].

By assessing the density of CD3^+^ T cells and CD8^+^ T cells in the tumor area through WSI, Lin et al*. *[41] established an automatic assessment model of I-score cell density that can be utilized for clinical risk prediction, demonstrated that a high immune infiltration rate of TME was related to a favorable prognosis of NSCLC.

In a separate investigation by Wang et al*.*, researchers attempted to address the unclear boundaries in conventional pathological image recognition by refining the method for identifying cell boundaries. Then, they proceeded to segment and classify cell nuclei, while representing blood vessels and necrosis using images of red blood cells and nuclear lysis. Finally, 48 features related to the cellular spatial organization were extracted and combined with the National Lung Screening Trial (NLST) dataset to develop a prognostic model, which was validated in TCGA. It was demonstrated that the predicted survival rate of the high-risk group was significantly lower than that of the low-risk group (*p* = 0.001) [42].

Park et al*. *[43] developed the TIL spatial analysis model Lunit SCOPE IO, which can segment and quantify tissue components in WSI images and was successfully utilized to predict the benefit of ICIs in patients with advanced NSCLC. Presently, numerous studies have used pathological image analysis to demonstrate the correlation of TMB, CD8^+^ T cells, regulatory T (T-reg) cells, and TILs with PD-1 therapy [29].

### Multi-omics-based AI in TME prediction

Just like PD-L1 and TMB, the compositional changes of TME are influenced by various factors [44]. Therefore, several studies have focused on multi-omics to predict TME.

In a recent study, experts used unsupervised cluster analysis with tumor immune microenvironment (TIME) data and survival data of 1906 adenocarcinoma patients. The resulting TIME score scoring model is characterized by significantly mutated genes (SMG), copy number variation (CNV), and cancer stemness. This model distinguishes immune infiltration and effectively predicts the sensitivity of immunotherapy and the accuracy of prognosis [44].

As is known to us, immunotherapy resistance may relate to dysregulated lactic acid metabolism that inhibits dendritic cell (DC) maturation, thereby minimizing T cell infiltration. A study screened a gene map associated with lactate metabolism with ML and verified the effectiveness of the seven screened genes related to lactate metabolism in predicting survival and immunotherapy efficacy in a cohort of adenocarcinoma patients [45].

Costimulatory molecules play a vital role in activating immune cells. However, the characterization of the many co-stimulatory molecular genes (CMGs) in LUAD is poorly understood. Thus, some scholars estimated the composition of stroma and immune cells in malignant tumor tissues through K-means clustering. They used SVM to screen out the CMG (CD80, LTB and TNFSF8) as the final markers to predict the TIME status of patients, achieving the purpose of predicting the effect of immunotherapy [46]. IHC verification of 16 specimens revealed a significant positive correlation between the screened biomarkers and the response to immunotherapy.

Taken together, these studies underscore the potential value of genomics in predicting TME and offer a promising direction for future research.

## The applications of AI and ML in lung cancer immunotherapy prediction and adverse effects

**Fig. 4:**
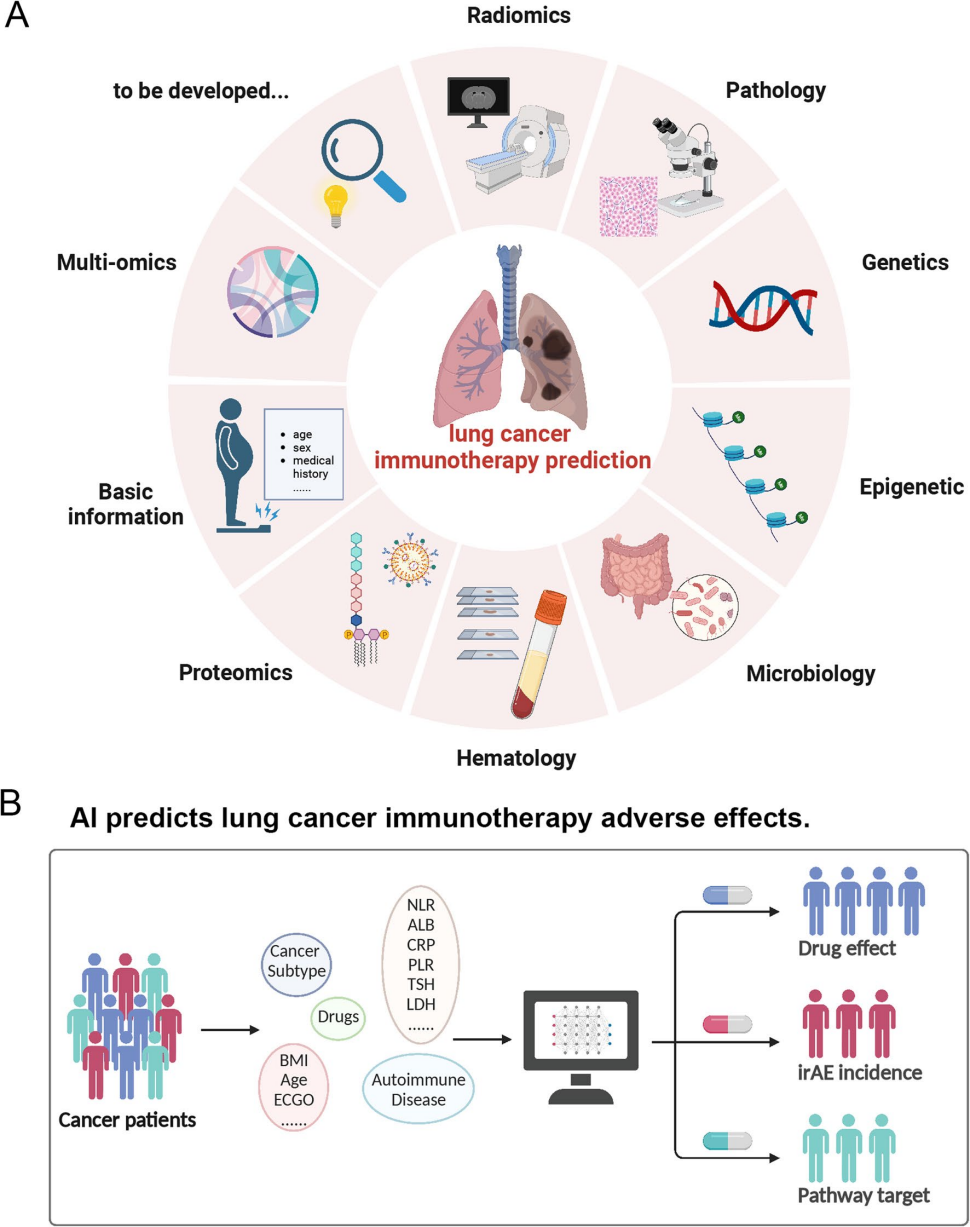
**A** Methods of lung cancer immunotherapy prediction. The application of AI-based technologies in lung cancer immunotherapy can process radiomics images, pathology images, genetics information, epigenetic information, microbiology information, hematology values, proteomics information, multi-omics data and so on. AI can use diverse data to predict immunotherapy benefits in lung cancer patients. **B** Al predicts lung cancer immunotherapy adverse effects. Abbreviation: irAEs: immune-related adverse events; BMI: Body mass index; ECOG PS: Eastern Cooperative Oncology Group performance status; NLR: Neutrophil to lymphocyte ratio; ALB: Albumin; PLR: Platelet-to-lymphocyte ratio; TSH: Thyroid-stimulating hormone; LDH: Lactate dehydrogenase. Created with BioRender.com

The prediction of therapy efficacy can be classified into direct predictions and indirect predictions. Common approaches such as radiomics, pathomics, and genomics can indirectly predict the relationship between PD-L1, TMB, and other biomarkers with survival and therapy efficacy. Conversely, proteomics and laboratory inspection data are mainly utilized for direct predictions (Fig. 4).

### Radiomics-based AI in immunotherapy prediction

Similar to the utilization of radiomics for the prediction of other biomarkers, when utilizing image data to predict the efficacy of immunotherapy, the majority of studies rely on feature extraction, amalgamated with immunotherapy data, and modeled with a range of ML algorithms. Although adjuvant therapy such as immunotherapy can control the progression of lung cancer patients to a large extent and prolong patients’ survival time with subsequent radiotherapy, chemotherapy, and surgery, there are still many patients who do not benefit from immunotherapy or are not suitable for surgery.

Clinical benefit such as progression-free survival (PFS) is the top predictive value for radiomics-based AI models. When utilizing durable clinical benefit (DCB, PFS ≥ 6 months) and non-DCB (NDCB, PFS < 6 months) as the endpoints to conduct predictive models, researchers found that weighted radiomics signatures of multiple intrapulmonary lesions have the potential to predict long-term PFS benefit in PD-1/PD-L1 immunotherapy candidates, which aggregates performance across all models and yields excellent results (AUCs: 0.75–0.82) [47].

Additionally, different algorithms were used to predict the immunotherapy efficiency or response rate. Tang et al*.* extracted image features from 422 NSCLC patients, assessing radiological parameters using DT, boosting tree (BT), RF, SVM, generalized linear model (GLM), and deep learning artificial neural network (DL-ANN). An AUC > 0.7 result was obtained for the prognostic performance of omics features. RF achieved an excellent performance of AUC = 0.938 among the models. This comparative study demonstrates the value of ML algorithms in the prediction process of immunotherapy. It is believed that with the iterative update of modeling methods, AI-assisted clinical decision-making will eventually become a reality [48]. Another study focuses on CT-based short-term follow-up radiomics features, utilizing an SVM model to predict the response to immunotherapy and PFS in patients with advanced NSCLC [10].

Moreover, Gong et al. used short-term follow-up CT images of patients for radiological histological features extraction, and performed a SVM model to predict the response of advanced NSCLC to immunotherapy and the PFS of patients [49]. Tian et al. analyzed a cohort of 939 patients with IIIB-IV NSCLC using a deep convolutional neural network (Deep CNN) algorithm to train the model "PD-L1ES". The test set results showed that PE-L1ES was able to predict high PD-L1 expression (PD-L1 ≥ 50%) with the AUC = 0.76. Meanwhile, a low PD-L1ES score was associated with the improvement of PFS (*p* = 0.010). When features commonly used in clinical prediction models such as age, gender, smoking history, and family history of malignant tumors are added to this prediction model, it can better predict the response of patients to immunotherapy and improve the stratification ability of the model [50]. The results showed that PD-L1 classification with DL features and quantitative radiomics features are complementary, which may be one of the directions to optimize the model performance in the future. Follow-up studies can incorporate more comprehensive immunotherapy patients’ information to improve the performance of the model in predicting the effect of immunotherapy, too.

Furthermore, the tumor and tumor organismal environment (TOE) features of pre-treatment CT images were extracted. SVM was used to make a risk stratification model. AUC = 0.869 was achieved in the validation set, which assists in predicting the differences in treatment response and survival outcomes in patients with standard locally advanced non-small cell lung cancer (LANSCLC) after radical concurrent chemoradiotherapy [51]. Moreover, Yan's team developed a detection model from the LUNA16 public database using DL. They performed detection on the Anti-PD-1_Lung dataset, comparing it with the effectiveness of immunotherapy, and ultimately demonstrating the ability of the model to predict immunotherapy in lung cancer [10]. Simultaneously, Mu et al*. *[4] collected PET-CT images before receiving ICI treatment, extracted features, and modeled them to predict clinical outcomes such as OS and PFS of patients.

In general, the research on radiomics to predict lung cancer immunotherapy efficacy is very popular and various algorithm types based on various characteristics have shown good model performance.

### Genomics-based AI in immunotherapy prediction

Changes in genetic material, epigenetic information, oncogenic and tumor suppressor signals, and transcription factors in tumors also influence the expression of PD-L1 in addition to TME. A reliable assessment of treatment efficacy requires accurate characterization of these compositions [30].

Immune regulation-related gene profiles in patient biopsy samples were analyzed by Wiesweg et al*. *[52] to develop an ML model to predict the impact of genomic information and TME on the therapeutic response for IV NSCLC.

DNA methylation (DNAm) or RNA methylation can serve as an epigenetic marker to predict cancer recurrence risk at a molecular level. ML algorithms were used to identify 4 CpG methylation markers associated with cell proliferation markers, somatic changes, TMB and DNA damage response (DDR) genes in a recent study. These markers were combined with clinical stage and survival data to construct a risk score model. And the model effectively predicted recurrence-free survival and prognosis of NSCLC patients (*p* = 0.0002). This 4-DNAm marker panel was useful for NSCLC prognosis, treatment decision-making and assessment of treatment response [53]. Similarly, Shang et al*.* developed MeImmS, a DNAm scoring system that accounts for differences in the methylation status of 8 CpG islands. They analyzed its correlation with T cell exhaustion, immune regulation, and immune cell activation. The researchers also confirmed that the combination of DNA methyltransferase inhibitors (DNMTi) and ICIs has a favorable effect on the outcome of NSCLC patients [54]. m6A-mediated immune genes were also developed in a prediction model by various ML algorithms. The model demonstrated its applicability to predict survival and distinguish patients' TME, genomic background, chemotherapy response and immunotherapy response propensity. It shows the potential of m6A modification in changing the TME of LUAD, participating in tumorigenesis as well as predicting efficacy [55].

Acetylation is also a common and reversible epigenetic alteration that plays a critical role in the initiation and progression of malignant tumors. However, the prognostic value of acetylation-related genes in early-stage LUAD remains unclear. Some scholars collected acetylation-related genes in the transcriptome of early LUAD patients in the TCGA database to try to identify the important biomarkers of early LUAD recurrence through differential analysis and protein–protein interaction network construction. Finally, they concluded that the two gene signatures of RBBP7 and YEATS2 can be used to predict the recurrence-free survival of early LUAD [56].

Currently, many studies attempt to filter gene combinations [57, 58], transcriptional profile information, blood microRNA, etc., as features for modeling. And these studies have exhibited the predictive performance not inferior to existing markers.

### Proteomics-based AI in immunotherapy prediction

Proteomics is an intuitive biomarker distinct from genomics for the reason that expressed proteins undergo post-translation modifications and interact directly with the host immune system and TME. Recently, mass spectrometry (MS) analysis of serum samples has been utilized to characterize independent proteome features and develop models for predicting clinical outcomes and side effects of patients [59].

Over 1,600 autoantibody biomarkers in serum were trained through an iterative unsupervised ML algorithm. Ultimately 13 were selected as features for modeling. The results showed that high expression of these features was associated with an overall 5-year survival rate of 7.6% in lung cancer patients, further highlighting the potential of serum proteomics in predicting survival [60].

### Microbiology-based AI in immunotherapy prediction

In recent years, research pertaining to the gut microbiome has garnered significant attention. Numerous studies have explored the correlation between the gut microbiome and the effectiveness of immunotherapy. However, there exists a dearth of models that predict the correlation between them.

Liu et al*.* conducted an analysis of gut microbiome samples from 79 NSCLC patients who underwent immunotherapy. The study utilized RF and multilayer perceptron (MLP) neural network models to predict PFS. Notably, the results indicated that two prediction models based on function rather than classification were both AUC ≥ 0.95. These outcomes suggest that the models can effectively predict the potential benefits of immunotherapy for NSCLC patients. Furthermore, it highlights the promising future of gut microbiome analysis in predicting cancer immunotherapy outcomes [52].

### Blood biomarkers-based AI in immunotherapy prediction

Due to the intricate mechanisms governing anti-tumor immune responses, conventional biomarkers are inadequate in dynamically illustrating tumor-immune system interactions and identifying patients who would benefit from ICI therapy [61]. Consequently, there is an urgent need to develop new markers. Recent research has focused on mining simple and readily available laboratory blood data. Some researchers collected medical history and laboratory examination data of NSCLC patients receiving ICI treatment and utilized various ML algorithms to model and screen predictive feature markers. Among these, the neutrophil to lymphocyte ratio (NLR) has exhibited predictive value for disease control rate (DCR) and 6-month survival in multiple studies [62, 63]. In addition to directly using routine inspection data, studies have employed techniques such as MS to obtain phenotypic characteristics of immune cells. By applying algorithms such as RF, partial least squares discriminant analysis, MLP and elastic network, scholars have discovered that B cell-related phenotypes can be used as features to distinguish healthy, responders and baseline non-responders and predict the ability to respond to immunotherapy [64].

Furthermore, another study employed 10 ML algorithms to screen data from three NSCLC immunotherapy cohorts for TMB, intratumoral heterogeneity and loss of heterozygosity for human leukocyte antigen. By combining these three genomic biomarkers using the SVM-poly method, a model was constructed to predict DCB, with the model exhibiting an AUC of 0.78 [61]. Expanding the training samples in such models is expected to enable the development of additional biomarkers, providing more personalized treatment options and promoting precision medicine.

### AI in lung cancer immunotherapy adverse effects prediction

Approximately one-third of patients who received immunotherapy experienced irAEs [65]. The progression of irAEs is the primary reason for discontinuing immunotherapy. Meanwhile, irAEs are a significant factor that affects the prognosis of patients [66, 67]. Currently, there are no biomarkers that can predict the early occurrence of irAEs, and few studies have been conducted in this regard. Therefore, creating a painless, accurate and standardized prediction method is a considerable challenge that requires further research [68].

Neural network models were utilized to predict skin irAEs caused by PD-L1 therapy considering variables such as tumor type, treatment drug, age, autoimmune history, derived NLR, lactate dehydrogenase, albumin, body mass index, Eastern Cooperative Oncology Group performance status (ECOG PS) and tumor M-stage features. The results demonstrated that ML has a high sensitivity and ability to predict cutaneous irAEs in the early stages of immunotherapy, with a positive predictive value (PPV) of 76.5% (± 10.5%), a negative predictive value (NPV) of 79.4% (± 11.9%), a sensitivity of 85.3% (± 8.8%), and a specificity of 67.6% (± 15.8%) [67].

Cardiac irAEs could be deadly. XGBoosted decision tree was used to predict the probability of cardiac adverse events in patients receiving PD-1 or PD-L1 therapy by modeling 356 basic medical history information as potential risk factors. In this study, 4,960 patients receiving PD-1/PD-L1 therapy were included, among whom 418 experienced cardiac events. The final model showed that age, corticosteroids, heart disease history drugs, extreme body weight, low lymphocyte percentage and high neutrophil percentage were associated with the occurrence of cardiac adverse events, but the model had limited predictive value with an AUC of 0.65 [69].

To predict adverse events in atezolizumab-treated advanced NSCLC patients, seven ML methods were employed to explore the role of 21 blood markers. XGBoost and LASSO methods performed the best, and the AUC of XGBboost for 10 markers was 0.692. However, even after narrowing down to the combination of C-reactive protein (CRP), platelet-to-lymphocyte ratio (PLR), and thyroid-stimulating hormone (TSH), the predictive effect was still not satisfactory, despite the high consistency between the training set and the test set [68].

It is evident that the results of irAE prediction studies have been unsatisfactory, and the biomarkers screened out by ML are just primarily related to adaptive immunity, inflammatory state, liver and thyroid function. Although these indicators correspond to clinical symptoms such as fatigue, vomiting and elevated transaminases, their abnormality already signifies that irAEs are in progress, rendering them of little predictive value. Therefore, exploring inflammatory pathway targets of different types of irAEs from a mechanistic perspective and realizing early prediction and targeted therapy of irAEs are future research directions (Fig. 4).

## Discussion and prospect

Although using ML to predict clinical information is currently a hot research topic, it is still in its early stages. It’s an innovative and cooperative attempt between computer experts and clinical doctors which has not yet been fully established. Due to practical reasons such as the non-sharing of cohort data and insufficient sample sizes, the models can only achieve good performance in internal validation sets, making it difficult to generalize and practice in real-world clinics.

During the pathology images training process, supervised learning requires pathologists to manually label features, which is difficult to apply to the entire gigapixel image. Therefore, it is necessary to optimize the DL framework and weaken the supervision mechanism to achieve high-throughput sample training [70].

Currently, many predictive models based on ML algorithms have emerged due to the development and popularization of digital images worldwide. Each model or algorithm has its strength and weakness (Table 3). However, the predictive performance of a single marker is mostly not ideal. Therefore, it is important to comprehensively use various features such as PD-L1, TMB, TME, miRNA, immune genes, gut microbiome, radiomics, baseline data and other omics features to continuously refine and improve the algorithms. With the aid of AI, doctors and patients can receive personalized decision-making assistance.

**Table 3:** Comparison of different algorithms in lung cancer immunotherapy prediction

Model	Algorithm	Category	Strengths	Weaknesses	Example
DenseNet	CNN	Radiomics for TMB and survive prediction	Available for better performance with fewer parameters and computational costs by dense connection and feature reuse	Worse performance than other algorithms under the same video memory usage	[5]
SResCNN	CNN	Radiomics for PD-L1 and survive prediction	Alleviate the network degradation problem caused by layer deepening and increased the generalization ability of the network	Network layer redundancy Insufficient effective depth	[14]
RF	ML	Radiomics for PD-L1 and survive prediction	Less likely to overfit Suitable for uneven data sets with missing variables Easier to explain Higher accuracy	The larger the number of decision trees, the higher memory usage. Not suitable for situations with high real-time requirements	[22]
Lunit SCOPE IO	DNN	Pathology images for TIL and prognosis prediction	Extracting richer data features and larger capacity	Training process is difficult: gradient explosion, gradient disappearance, etc.	[43]
LCI-RPV	LR	Multi-omics for PD-L1 and Pneumonia prediction	Suitable for linear variables Easier to explain	Difficult to process nonlinear data or polynomial regression with correlation between data features	[20]
MLP	ANN	Gut microbiome for survive prediction	Suitable for nonlinear model and real-time learning process Stronger elf-learning function	Slower training rate Difficult to determine the parameters	[52]
SVM	ML	Combined biomarkers for efficiency prediction	Suitable for high-dimensional space High accuracy Not suffer multicollinearity Flexible selection of kernels for nonlinear correlation	Inefficient to train Not suitable for plenty training examples	[46]

*DenseNet* Densely Connected Convolutional Network, *SResCNN* Small Residual Product Network, *CNN* Convolutional Neural Network, *TMB* Tumor Mutation Burden, *PD-L1* Programmed Death Ligand 1, *RF* Random Forests, *ML* Machine Learning, *DNN* Deep Neural Networks, *ANN* Artificial Neural Network, *MLP* Multilayer Perceptron, *SVM* support vector machine

With the development of lung cancer screening, accurately judging the benign or malignant characteristics of pulmonary nodules or ground-glass opacities is the key point for early screening. Even experienced radiologists are also prone to misdiagnosis. As a computer-aided diagnosis (CAD) system developed earlier and with better performance, the CT-assisted image reading tool has been already applied in some hospitals to identify, classify and qualitatively evaluate the malignancy of pulmonary nodules and predict treatment response through AI [71]. Its auxiliary diagnostic value has been clinically recognized.

In the future, AI-based radiomics may solve the limited predictive performance of the model through code and case sharing to increase the universality of the model and the possibility of entering into clinical applications. At the same time, the application of radiomics images is no longer limited to predicting the expression levels of immunotherapy biomarkers. At present, there are a few studies that combined deep learning models with reinforcement learning algorithms to improve and optimize algorithms, using 3D reconstructed images to accurately locate, segment, and classify tumors [72]. Perhaps in the future, AI-based radiomics can not only use images of diagnosed lung cancer patients for curative effect evaluation and survival prediction but also analyze image abnormalities in physical examination populations, thus making a better function in lung cancer’s early screening and early diagnosis [73].

For the challenge of AI in lung cancer pathological images, pathologists are required to label more regions and more types of massive images for AI to optimize performance [74]. However, for disease changes that cannot be qualitatively described, AI still cannot replace manual diagnosis.

While using Formalin-Fixed Paraffin-Embedded (FFPE) sections for routine immunohistochemical staining to give a diagnosis, some researchers have tried to push multiplex immunohistochemistry (mIHC) staining into the clinic, using automated equipment to obtain images of the co-expression of tumor cells and immune cells. Then, the immune infiltration situation is reconstructed in 3D and the multi-dimensional data are analyzed by AI, which can better evaluate the TIL status of patients and predict the benefit ratio of immunotherapy. However, this technology has not been widely promoted from the scientific research level to clinical trials, and there is still a lack of unified norms and expert consensus. At the same time, due to the high cost and high technical requirements of pathological image scanners and analysis software, the path for AI to replace pathologists for the diagnosis will be even longer.

However, AI has made some progress in the early diagnosis and screening of lung cancer, pathological classification, immunotherapy efficacy and prognosis evaluation. It is believed that with the deepening of research, AI will bring more benefits to lung cancer patients in the future.

Although ICIs have made great breakthroughs in cancer treatment, the low proportion of beneficiaries based on PD-1/PD-L1/CTLA-4, the high proportion of irAEs, and the high cost of treatment are the main problems to be solved urgently in current immunotherapy limited [75]. AI can not only predict the beneficiary population and reduce the proportion of adverse events through the joint use of markers but also use big data to develop new types of markers. Clarifying the mechanism of irAE is also the key to solving the problem. At the same time, it needs to be ascertained whether the poor effect of immunotherapy is related to the drug delivery approaches [76, 77].

Meanwhile, the development of new ICIs is imperative. The number of approved cancer treatment drugs based on ICIs has been increasing, and it is also an enduring research hotspot [78]. Immunotherapy drugs targeting T cell immunoreceptors with immunoglobulin and ITIM domains, the lymphocyte activation gene 3, T cell immunoglobulin, mucin-domain 3 and immune checkpoint siglec-15 are in clinical trials or under development [74, 79].

To conclude, AI combines, disassembles, and analyzes data in a "black box" manner, showing great promise in the predictive application of cancer immunotherapy. It is believed that with the rapid development of science and technology, more people will benefit from the application of artificial intelligence in medical treatment.

## Data Availability

All data are available in the main text or the supplementary materials.

## Abbreviations

ML: Machine learning
AI: Artificial intelligence
PD-L1: Programmed death-ligand 1
PD-1: Programmed cell death protein 1
TMB: Tumor mutation burden
TME: Tumor microenvironment
DL: Deep learning
ICB: Immune checkpoint blockade
UL: Unsupervised learning
SSL: Semi-supervised learning
KNN: K-nearest neighbor
LR: Linear regression
SVM: Support vector machine
DT: Decision tree
RF: Random Forest
PCA: Principal component analysis
CV: Computer vision
NLP: Natural language processing
CT: Computer tomography
PET/CT: Positron emission tomography/computer tomography
IHC: Immunohistochemistry
ANN: Artificial neural network
CVPR: Conference on computer vision and pattern recognition
CNN: Convolutional neural network
DNN: Deep neural network
ICI: Immune checkpoint inhibitor
CTLA-4: Cytotoxic T lymphocyte-associated antigen-4
NSCLC: Non-small cell lung cancer
irAEs: Immune-related adverse events
WES: Whole exome sequencing
OS: Overall survival
NCCN: National comprehensive cancer network
NB: Naive Bayes
AUC: Area under the curve
SResCNN: Small-residual-convolutional-network
DLS: Deep learning score
ROC: Receiver operating characteristic curve
QVT: Quantitative vessel tortuosity
WSI: Whole slide image
TIL: Tumor infiltrating lymphocyte
TPS: Tumor proportion score
TCGA: The Cancer Genome Atlas
qRT-PCR: Quantitative real-time-polymerase chain reaction
sCEA: Serum CEA
CAF: Cancer-associated fibroblast
GGO: Ground-glass opacity
pGGO: Pure ground-glass opacity
LUAD: Lung adenocarcinoma
GEO: Gene Expression Omnibus
NLST: National Lung Screening Trial
T-reg cells: Regulatory T cells
TIME: Tumor immune microenvironment
SMG: Significantly mutated gene
CNV: Copy number variation
DC: Dendritic cell
CMG: Costimulatory molecular gene
PFS: Progression-free survival
DCB: Durable clinical benefit
NDCB: Non-durable clinical benefit
BT: Boosting tree
GLM: Generalized linear model
DL-ANN: Deep learning artificial neural network
Deep CNN: Deep convolutional neural network
TOE: Tumor organismal environment
LANSCLC: Locally advanced non-small cell lung cancer
DNAm: DNA methylation
DDR: DNA damage response
DNMTi: DNA methyltransferase inhibitor
MS: Mass spectrometry
MLP: Multilayer perceptron
NLR: Neutrophil to lymphocyte ratio
DCR: Disease control rate
ECOG PS: Eastern Cooperative Oncology Group performance status
PPV: Positive predictive value
NPV: Negative predictive value
CRP: C-reactive protein
PLR: Platelet-to-lymphocyte ratio
TSH: Thyroid-stimulating hormone
CAD: Computer-aided diagnosis
FFPE: Formalin-fixed paraffin-embedded
mIHC: Multiplex immunohistochemistry
